# Expanding the Clinical and Mutational Spectrum of Recessive *AEBP1*-Related Classical-Like Ehlers-Danlos Syndrome

**DOI:** 10.3390/genes10020135

**Published:** 2019-02-12

**Authors:** Marco Ritelli, Valeria Cinquina, Marina Venturini, Letizia Pezzaioli, Anna Maria Formenti, Nicola Chiarelli, Marina Colombi

**Affiliations:** 1 Division of Biology and Genetics, Department of Molecular and Translational Medicine, University of Brescia, 25123 Brescia, Italy; marco.ritelli@unibs.it (M.R.); v.cinquina@studenti.unibs.it (V.C.); l.pezzaioli001@studenti.unibs.it (L.P.); nicola.chiarelli@unibs.it (N.C.); 2 Division of Dermatology, Department of Clinical and Experimental Sciences, Spedali Civili University Hospital, 25123 Brescia, Italy; marina.venturini@unibs.it; 3 Spedali Civili of Brescia, 25123 Brescia, Italy; 4 IRCCS Istituto Ortopedico Galeazzi, 20161 Milano, Italy; annaformenti@live.it

**Keywords:** classical Ehlers-Danlos syndrome, classical-like Ehlers-Danlos syndrome type 2, *AEBP1*, aortic carboxypeptidase-like protein, differential diagnosis, high-frequency ultrasonography, reflectance confocal microscopy

## Abstract

Ehlers-Danlos syndrome (EDS) comprises clinically heterogeneous connective tissue disorders with diverse molecular etiologies. The 2017 International Classification for EDS recognized 13 distinct subtypes caused by pathogenic variants in 19 genes mainly encoding fibrillar collagens and collagen-modifying or processing proteins. Recently, a new EDS subtype, i.e., classical-like EDS type 2, was defined after the identification, in six patients with clinical findings reminiscent of EDS, of recessive alterations in *AEBP1,* which encodes the aortic carboxypeptidase–like protein associating with collagens in the extracellular matrix. Herein, we report on a 53-year-old patient, born from healthy second-cousins, who fitted the diagnostic criteria for classical EDS (cEDS) for the presence of hyperextensible skin with multiple atrophic scars, generalized joint hypermobility, and other minor criteria. Molecular analyses of cEDS genes did not identify any causal variant. Therefore, *AEBP1* sequencing was performed that revealed homozygosity for the rare c.1925T>C p.(Leu642Pro) variant classified as likely pathogenetic (class 4) according to the American College of Medical Genetics and Genomics (ACMG) guidelines. The comparison of the patient’s features with those of the other patients reported up to now and the identification of the first missense variant likely associated with the condition offer future perspectives for EDS nosology and research in this field.

## 1. Introduction

Ehlers-Danlos syndrome (EDS), with an estimated prevalence of 1/5000, comprises a group of clinically heterogeneous heritable connective tissue disorders (HCTDs) with diverse molecular etiologies. The 2017 revised EDS classification recognized 13 distinct subtypes caused by pathogenic variants in 19 genes mainly encoding fibrillar collagens, collagen-modifying proteins, or processing enzymes [1]. Classical EDS (cEDS) (MIM #130000), with an estimated prevalence of 1/20,000, is an autosomal dominant disorder primarily characterized by cutaneous and articular involvement. Indeed, cEDS is suggested by skin hyperextensibility plus atrophic scarring that must be present together with the other major criterion, i.e., generalized joint hypermobility (gJHM) evaluated according to the Beighton score (BS ≥5/9), and/or with at least three of the minor criteria among easy bruising, soft, doughy skin, skin fragility, molluscoid pseudotumors, subcutaneous spheroids, hernia (or a history of thereof), epicanthal folds, JHM complications (e.g., sprains, luxation/subluxation, pain, flexible flatfoot), and family history of a first-degree relative who meets clinical criteria [1,2,3]. Furthermore, cEDS patients may present distinctive facial features, premature rupture of fetal membranes, scoliosis, osteoporosis, gastroesophageal reflux, and cardiac and blood vessel fragility [2,4,5,6,7,8]. Skin is hyperextensible if it can be stretched over a standardized cut off in the following areas: 1.5 cm for the distal part of the forearms and the dorsum of the hands; 3 cm for neck, elbow and knees; 1 cm on the volar surface of the hand (palm) [1,2,5]. Atrophic scarring can range in severity; however, most cEDS patients have wide atrophic scars in different body areas that can variably assume a cigarette paper, papyraceous, or hemosiderotic appearance [1,2,5].

Point mutations or intragenic rearrangements of the *COL5A1* and *COL5A2* genes encoding type V collagen are recognized in over 90% of patients [4,9], the recurrent heterozygous *COL1A1* c.934C>T (p.Arg312Cys) substitution is rarely found [4,10,11]. Negative molecular testing does not exclude the diagnosis, as specific types of mutations (e.g., deep intronic variants) may go undetected by standard diagnostic molecular techniques. Nevertheless, alternative diagnoses should be taken into account in the absence of a *COL5A1, COL5A2,* and *COL1A1* mutation [1].

Recognition of cEDS is straightforward in the patient with the typical cutaneous signs and BS ≥5. However, intra- and interfamilial variability tells a much broader clinical presentation and significant overlap with other EDS types and HCTDs [1,2,4,5,6,12]. Differential diagnosis of cEDS should include the hypermobile EDS (hEDS), particularly in patients without a striking cutaneous involvement [1,12]. Indeed, hEDS shares with cEDS gJHM and many mucocutaneous signs, but generally a lower grade of skin hyperextensibility and only few small atrophic or post-surgical enlarged scars are observed [13,14]. In case of a family history compatible with autosomal recessive transmission, differential diagnosis comprises the rare classical-like EDS type 1 (MIM #606408) due to biallelic *TNXB* mutations. These patients show marked skin hyperextensibility, easy bruising, and joint laxity, but unlike cEDS patients, they do not have atrophic scarring or poor wound healing. Furthermore, minor criteria such as foot deformities, edema in the legs, mild proximal and distal muscle weakness, axonal polyneuropathy, and atrophy of muscles in hands and feet facilitates the differential [1,11]. Severe progressive cardiac-valvular problems distinguish the cardiac-valvular EDS type (*COL1A2*) from cEDS, severe skin fragility and unusual craniofacial features discriminates the dermatosparaxis EDS (*ADAMTS2*), whereas (congenital) kyphoscoliosis and muscle hypotonia differentiates the kyphoscoliotic EDS (*PLOD1*, *FKBP14*), which are other rare recessive EDS types. Bilateral congenital hip dislocation differentiates the autosomal dominant arthrochalasia EDS (*COL1A1*, *COL1A2*) [1,11,12].

Recently, in six individuals from four unrelated families who presented with a constellation of clinical findings reminiscent of cEDS such as gJHM, redundant and hyperextensible skin with poor wound healing and abnormal scarring [15,16,17], and recessive alterations in the *AEBP1* gene, which encodes the aortic carboxypeptidase-like protein (ACLP) associating with collagens in the extracellular matrix, were recognized, thus defining a new EDS form labelled as classical-like EDS type 2 (MIM #618000).

Herein, we describe an additional patient with a homozygous missense *AEBP1* causative variant and compare her clinical features with those of the other patients reported so far, offering future perspectives for EDS nosology and research in this field.

## 2. Patient and Methods

### 2.1. Molecular Analyses

The patient was evaluated at the specialized outpatient clinic for the diagnosis of EDS and related connective tissue disorders, i.e., the Ehlers-Danlos Syndrome and Inherited Connective Tissue Disorders Clinic (CESED), at the University Hospital Spedali Civili of Brescia. Molecular analysis was achieved in compliance with the Italian legislation on genetic diagnostic tests and the patient provided written informed consent for publication of clinical data and photographs according to the Italian bioethics laws. Since this report is based on data obtained through routine clinical care and is not considered research at the involved institutions; formal ethics review was not obtained. Genomic DNA was extracted from peripheral blood leukocytes using standard procedures; the exons and intron-flanking regions of *COL5A1*, *COL5A2*, and exon 14 of *COL1A1* (c.934C>T (p.Arg312Cys) were amplified by PCR and directly sequenced using an ABI PRISM^®^ 3130XL Genetic Analyzer (Life Technologies, Carlsbad, CA, USA), as previously reported [4]. For the multiplex ligation-dependent probe amplification (MLPA), the commercially available SALSA MLPA kits P331 and P332 for *COL5A1* gene were used, according to the manufacturer’s recommendations (MRC-Holland, Amsterdam, The Netherlands), as previously described [4]. The primers for *AEBP1* Sanger sequencing (Supplementary Table 1) were designed for all coding exons, including the intron-exon boundaries, and primer sequences were analyzed for the absence of known variants using the GnomAD database [18]. The sequences were analyzed with the Sequencher 5.0 software and variants were annotated according to the Human Genome Variation Society (HGVS) nomenclature by using the Alamut Visual software version 2.11. To evaluate the putative pathogenicity of the *AEBP1* missense variant, which was submitted to the LOVD Ehlers–Danlos Syndrome Variant Database [19], we used the following mutation prediction programs: Mutation Assessor [20], PhD-SNP [21], Align GVD [22], SIFT [23], Mutation Taster [24], PolyPhen2 [25], PROVEAN [26], MutPred [27], M-CAP [28], CADD [29], DANN [30], Fathmm-MKL [31], and VEST [32]. The nucleotide and protein accession numbers correspond to the *AEBP1* (NM_001129.4, NP_001120.3) reference sequences.

### 2.2. High-Frequency Ultrasonography and In Vivo Reflectance Confocal Microscopy

To investigate patient’s skin by a non-invasive approach, we performed high-frequency ultrasonography (HF-USG) and *in vivo* reflectance confocal microscopy (RCM) as previously described [33,34,35].

Briefly, HF-USG was performed on the dorsal and volar side of the forearm of the patient and 10 age- and gender-matched healthy individuals with the same skin phototype and similar sun exposure history by digital 50-MHz ultrasonography B mode scanning (DUB-USB Skin Scanner, Taberna Pro Medicum Company, Lueneburg, Germany). For ultrasound transmission, water was employed as a coupling medium between the transducer and the skin surface. The usable depth of signal penetration was 4 mm, and the gain was 40 dB. Ultrasonography images were collected under standard conditions (environmental temperature was 20–23 °C and the patient remained in a lying position for at least 10 min before examination). Acquired images were exported into a dedicated database and were evaluated using specific image-analysis software to assess epidermal and dermal thickness (µM) and lesional echogenicity.

RCM investigation on the same sides of the forearm was achieved with a Vivascope 1500^®^ microscope (MAVIG GmbH, Lucid Technologies, Henrietta, NY, USA) to visualize *in vivo* the horizontal optical skin sectioning at cellular-level resolution (lateral resolution = 0.5–1 µM, axial resolution = 3–5 µM) from the epidermis to the papillary dermis (200–250 µM in depth). The system uses a laser source with a wavelength of 830 nm and a power <35 mW at the tissue level. The microscope objective is attached to the skin through an adhesive ring to diminish motion artefacts during investigation. Water was used between the adhesive window and the skin, and ultrasound gel (Aquasonic 100 Gel; Parker Laboratories Inc., Fairfield, NJ, USA) was used between the adhesive window and the lens as detailed in [35]. VivaScan 7.0, Viva Stack^TM^ and Viva Block^TM^ software (Lucid Technologies, Henrietta, NY, USA) was employed to acquire blocks of 4 × 4 mm horizontal optical sections, obtained from 64 individual horizontal optical sections (500 × 500 µm images). The system saves images in bitmap format with digital resolution of 1000 × 1000 pixels and 256 levels of grey.

## 3. Results

### 3.1. Clinical Findings

**Figure 1:**
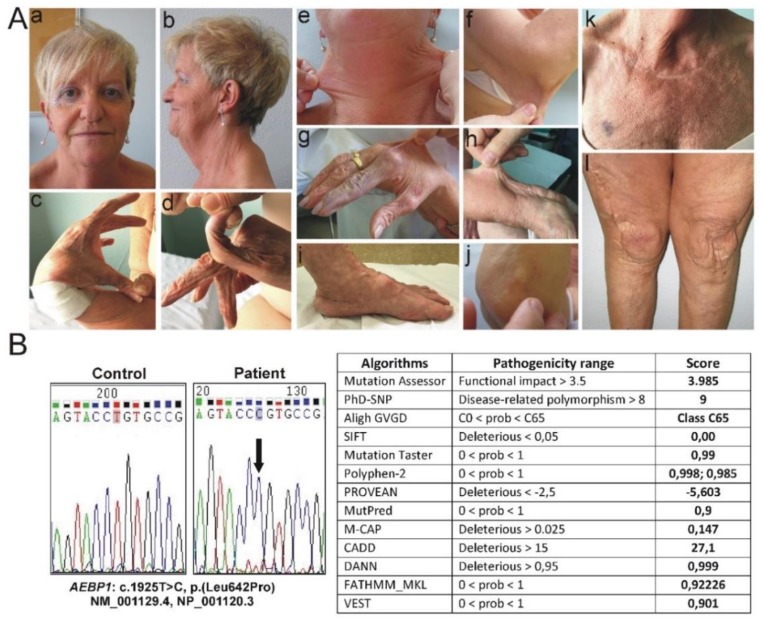
Clinical and molecular findings of the patient. (**A**) Old-aging appearance of face and androgenetic alopecia (a,b); laxity of the thumb (c), laxity of the fifth finger (d); hyperextensible skin in different body areas: neck (e), elbow (f), dorsum of the hand (g) and forearm (h); flat feet and piezogenic papules (i); subcutaneous spheroid on elbow (j), diffuse PoC-like dermatitis and easy bruising (k); skin redundancy, atrophic papyraceous scars on knees, postsurgical enlarged scar after right knee capsuloplasty and skin plastic surgery (l). (**B**) Sequence chromatograms showing the position of the c.1925T>C p.(Leu642Pro) variant (arrow) identified in homozygosity in exon 16 of the *AEBP1* gene (seq. Ref.: NM_001129.4, NP_001120.3) and *in silico* prediction of the pathogenicity of the p.(Leu642Pro) missense substitution by using 13 different algorithms [20,21,22,23,24,25,26,27,28,29,30,31,32].

The proband (LOVD ID AN_006205) was an Italian 53-year-old woman, born from healthy second-cousins parents, and had two healthy brothers. Clinical history was remarkable for premature birth at 30 weeks (height 44 cm, weight 1.2 kg) associated with perinatal respiratory distress. Neonatal severe hypotonia and delayed motor development, i.e., delays in walking (she took her first steps at four years of age) and acquisition of fine motor skills, were also reported. Medical history further included propensity to develop ecchymoses either spontaneously or upon minimal trauma often occurring for motor clumsiness, surgically treated umbilical hernia in infancy, myopia and astigmatism since childhood, and complete dental loss due to unspecified periodontitis at 14 years old. At age 18, a clinical diagnosis of unspecified EDS was given for gJHM, skin hyperextensibility, delayed wound healing, and easy bruising; genetic analyses were not performed. The patient suffered from recurrent dislocations of knees and occasionally of shoulders and elbows since the age of 10; the objective patellar instability was surgically treated by capsuloplasty and transposition of the insertion of the common patellar tendon by tibial tuberosity transplantation followed by skin plastic surgery at the age of 29 leading to a wide atrophic post-surgical scar (Figure 1A). At 21 and 23 years old, respectively, she underwent bilateral saphenectomy for symptomatic varicosities with pain, fatigability, heaviness, and recurrent superficial thrombophlebitis and surgical removal of nodules on vocal cords. At age 41, the patient was subjected to operative treatment of rotator cuff disease in the setting of weakness and substantial functional disability. Since age 42, she suffered from Achilles tendinopathy with pain and stiffness, especially at the back of the ankle, treated with on-demand NSAIDs use, conservative physical therapy, and orthotic insoles for severe pes planus. At 43 years of age, metatarsal osteotomy on the 3rd toe of the right foot for metatarsalgia and aggravating Achilles tendinopathy was performed. In the same period, she developed disabling bilateral gonarthrosis, treated with arthroscopic abrasion, epitrochleitis, and subacromial shoulder impingement associated with night pain. Hypotrophy of the scapular girdle and weak osteotendinous reflexes were observed at age 50, when she also experienced the dislocation of the left ankle with soft tissue effusion without reabsorption.

On examination, at 52 years of age, she presented with a height of 150 cm (genetic target 157 cm, arm span/height ratio 1.03, normal value <1.05), a weight of 52 kg, hyperextensible, soft, doughy, fragile and redundant skin, with an old-aging appearance of face and extremities, and multiple atrophic papyraceous scars, especially on knees, defective wound healing, easy bruising, spheroids on the elbows, and BS 5/9 (Figure 1A). She also showed multiple papules with some follicular prominence that looked like a diffuse poikiloderma of Civatte (PoC-like dermatitis) more pronounced in photo-exposed sites, androgenetic alopecia, high palate, elongated uvula, scoliotic attitude, mobile patellae and flat feet (even though surgical intervention and orthotics, respectively), hallux valgus, bilateral piezogenic papules, peripheral artery disease (i.e., intermittent claudication, peripheral cyanosis, and cold skin), and varicose veins (Figure 1A). The patient reported persistent lumbar back pain and sporadic pain of hips, knees, left ankle, elbows, shoulders, and feet. Multidimensional fatigue inventory (MFI) questionnaire was suggestive for chronic fatigue (total score 69, higher score in the questions investigating physical fatigue). Cognitive development and mentation were normal. Heart ultrasound detected normal cardiac/valve morphology and function. Dual-energy X-ray absorptiometry (DXA) disclosed femoral osteopenia (T-score left femoral neck −1.5 SD, T-score total hip −1.6 SD); lumbar BMD was normal (T score −0.9 DS) in the presence of marked degenerative arthritis. Nevertheless, we found a mild dorsal vertebral deformity (T10) in the presence of a low TBS value (1.23). The patient also presented mild scoliosis and lumbar spine rectilinization. Due to hypovitaminosis D, Cholecalciferol 50,000 UI monthly was commenced. Other bone metabolism blood and urinary samples and markers of bone remodeling were normal.

### 3.2. Molecular Findings

The patient’s phenotype was suggestive for cEDS, since she fulfilled both major (skin hyperextensibility plus atrophic scarring and gJHM) and 6 minor criteria according to the 2017 EDS nosology, i.e., easy bruising, soft, doughy skin, skin fragility, subcutaneous spheroids, a history of hernia, and JHM complications. Therefore, after written informed consent was obtained, we performed Sanger sequencing of *COL5A1*, *COL5A2*, and of exon 14 of *COL1A1* (p.Arg312Cys), integrated by MLPA analysis of *COL5A1*, which did not identify any pathogenic variant. Although negative molecular testing, a clinical diagnosis of cEDS was maintained, since the other EDS types in differential diagnosis with cEDS (including periodontal EDS) were excluded clinically. Following the discovery of *AEBP1* biallelic variants [15,16], Sanger sequencing of this gene was achieved, which revealed the homozygosity for the rare c.1925T>C p.(Leu642Pro) variant in exon 16 (Figure 1B), leading to the substitution of a highly conserved leucine residue with a proline at position 642 within the metallocarboxypeptidase-like domain of the protein. This variant has been observed in 3 individuals in GnomAD (rs753531562, 3/282140, no homozygotes, total MAF: C = 0.00001063). Its putative pathogenicity was estimated through an array of 13 different *in silico* prediction algorithms that agreed to define p.(Leu642Pro) as high impacting variant. Given that the variant is located in a critical and well-established functional domain without benign variation, the extremely low frequency in publicly available population databases, the multiple lines of computational evidence supporting a deleterious effect on the gene product, and the patient’s phenotype highly suggestive for a disease with a single genetic etiology, the p.(Leu642Pro) missense variant is classified as likely pathogenic (class 4) according to the guidelines of the ACMG. Samples of the healthy parents or brother were not available for molecular analyses.

### 3.3. Instrumental Findings on Patient’s Skin

**Figure 2:**
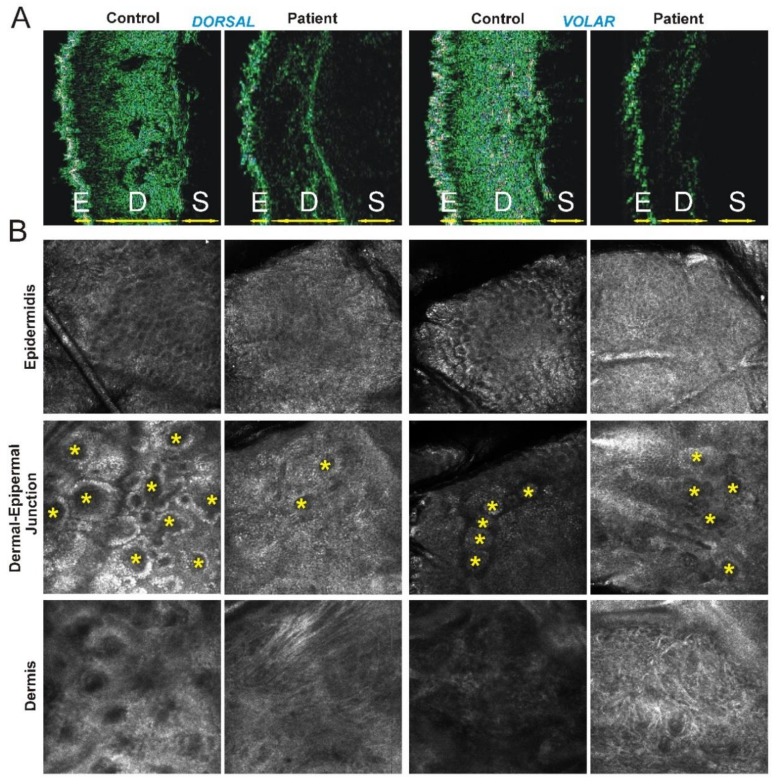
Instrumental findings on patient’s skin. (**A**) Ultrasonography (50 MHz) images of the forearm skin from the patient and a representative age- and gender-matched healthy individual (control). E, epidermis, D, dermis, S, subcutaneous adipose tissue (depth of imaging: 4 mm). Disorganization of collagen fibers and elastosis in patient’s skin appears as a significant thinning and hypoechogenicity of the dermal layer both on dorsal (left) and volar side (right) compared to control (**B**) Reflectance confocal microscopy images of the forearm skin from patient and control (magnification: 500 × 500 µm). Epidermis: typical honey-comb pattern on dorsal (left) and volar (right) side in healthy skin are not detectable in patient’s skin. Dermal-epidermal junction: regular edge papillae [rings of basal keratinocytes surrounding dark circular structures corresponding to dermal papillae (*)] on dorsal and volar side of control skin are reduced both in number and definition in patient’s skin. Dermis: Irregular and fragmented collagen fibers that appear bright and coarse on both dorsal and volar side of patient’s skin compared to control. An increased brightness of all skin structures, corresponding to chronological and photoinduced skin aging, is present on the dorsal side of healthy skin but not on the volar side that usually is not photoexposed, whereas in the patient this pronounced skin aging is present at both sides.

In order to investigate the skin by a non-invasive approach, HF-USG and *in vivo* RCM were performed on selected skin areas, i.e., dorsal and volar side of the forearm, showing clinically significant differences between our patient and 10 healthy individuals (Table 1 and Figure 2). Digital 50-MHz ultrasonography scanning demonstrated an increase in epidermal entrance echo (highly echogenic band produced by the differences of the acoustic impedance between gel and skin) corresponding to increased epidermal thickness, but a decrease in dermal thickness compared to control skin of age- and gender-matched healthy individuals with the same skin phototype II and similar sun exposure history. The patient’s epidermis (dorsal thickness = 172 µM; volar thickness = 141 µM) was thicker than that of healthy controls (dorsal thickness (mean ± standard deviation, SD) = 121 ± 22 µM; volar thickness (mean ± SD) = 102 ±12 µM), likely due to the multiple and diffuse papules (Table 1). The increased thickness was more evident on the dorsal side of the forearm that is chronically more photoexposed compared to the volar side. Contrariwise, the patient’s dermis appeared thinner (dorsal dermal thickness = 570 µM; volar epidermal thickness = 289 µM) compared to healthy controls (dorsal dermal thickness (mean ± SD) = 1108 ± 320 µM; volar epidermal thickness (mean ± SD) = 983 ± 205 µM) (Table 1). Moreover, the considerable hypoechogenicity of the dermal layer suggests disruption of collagen fibers and accumulation of elastotic material that is typical of chronological and photoinduced skin aging (Figure 2A). This ultrastructural pattern is known as subepidermal low echogenic band (SLEB) and derives from skin elastosis and accumulation of glycosaminoglycans that have increased water-binding capacity [33]. *In vivo* RCM investigation demonstrated loss of the typical honey-comb pattern (corresponding to alteration of epidermal thickness), irregularity of the dermal-epidermal junction and the disarray of the dermis, which was characterized by coarse and fragmented collagen fibers both on the dorsal and volar side of patient’s forearm (Figure 2B). These alterations are independent of sun exposure, given that they are present both on dorsal and volar side of the forearm, suggesting a pronounced and diffuse skin aging due to *AEBP1*-defect.

## 4. Discussion

Recently, taking advantage from NGS, a new, autosomal recessive type of EDS has been discovered due to variants in the *AEBP1* gene. This EDS type is very rare and, so far, found in only seven individuals (including the present patient) from four unrelated families (Table 2). The International Consortium on EDS and Related Disorders has not yet classified and named this type, but in OMIM it is labeled as classical-like EDS type 2 (MIM #618000). Indeed, the few patients reported hitherto (Table 2) share many similarities representative of the classical type as much as they all fulfill the cEDS diagnostic criteria of the 2017 nosology [1,2] for the presence of the pathognomonic cutaneous involvement, i.e., soft, doughy and very hyperextensible skin, delayed wound healing with abnormal atrophic scarring, JHM, and other minor criteria such as easy bruising, subcutaneous spheroids (observed only in our patient), and JHM complications such as dislocations/subluxations (shoulders, knees, hips, ankles, elbows, clavicula, wrist, mandibular and distal radioulnar joints, in some cases requiring surgical treatment), sprain, pain, and flexible flatfoot (Table 2)

Consistent with the multisystemic presentation of EDS in general, there are also variable features including congenital hip dislocation, hypotonia, delayed motor development, acrogeria, prominent superficial veins in the chest region, hernias, dental anomalies, gastrointestinal (bowel rupture) and vascular complications (mitral valve prolapse, aortic root dilation needing surgery), early-onset varicose veins, and several skeletal anomalies (Table 2). In particular, bone involvement seems a common feature of classical-like EDS type 2 with osteopenia/osteoporosis affecting hips, femurs, and spine that are present, at variable degree, in all of the patients reported so far, with the exception of the two siblings, reported by Hebebrand and coworkers [17], who were not tested for osteopenia. In addition, degenerative arthritis, (kypho)scoliosis, arachnodactyly, positive wrist and thumb signs, mild pectus excavatum, T10 vertebral deformity (our patient), narrowing of the interpedicular distance of the lumbar spine, shortened and squared iliac bones, and remodeling of long bones of the lower extremities are also encountered (Table 2). In addition, all subjects have severe foot deformities including bilateral pes planus, hammertoes, hallux valgus, hindfoot deformity, and sandal gap, which are observed in more than a few other EDS subtypes as well [1]. Although in cEDS patients a variable degree of low bone mineral density and a high prevalence of radiological vertebral fractures were reported [7,36], *AEBP1*-related EDS seems to display a more severe bone involvement that could potentially facilitate the differential with cEDS. Nevertheless, considering the limited number of individuals with *AEBP1* defect known so far, a larger cohort of patients is needed to confirm this preliminary observation.

The adipocyte enhancer binding protein 1 gene (*AEBP1)* encodes a 1158-amino acid secreted aortic carboxypeptidase-like protein (ACLP) composed of an N-terminal signal sequence, a charged lysine, proline, and glutamic acid-rich domain, a collagen-binding discoidin domain and a metallocarboxypeptidase (MCP)-like domain [37,38]. This latter domain is inactive toward standard MCP substrates, as it lacks several critical active sites and substrate-binding residues that are necessary for activity [37,38]. Indeed, ACLP acts as an extracellular matrix (ECM)-binding protein rather than as active MCPs that shows similar embryonic expression pattern as other ECM proteins and is found at high levels particularly in collagen-rich tissues comprising the dermal layer of the skin, the medial layer of blood vessels, the basement membrane of the lung, and the periosteum. Consistently, ACLP plays fundamental roles in both embryonic development and adult tissue homeostasis, particularly in repair processes [38,39,40,41,42,43]. Indeed, *AEBP1* knock-out mice show ventral wall defects, develop spontaneous skin ulcerations, and have significantly delayed healing of dermal punch wounds [38]. This cutaneous phenotype is consistent with the defective wound healing and abnormal scar formation observed in individuals with *AEBP1* defects and suggest that ACLP has a crucial role in damage sensing and ECM remodeling following injury by regulating fibroblast proliferation and mesenchymal stem cell differentiation into collagen-producing cells [42,43]. Blackburne and coworkers demonstrated that ACLP also binds collagens type I, III, and V and is able to promote the polymerization of collagen type I in vitro [16]. In line with these findings, the ultrastructural study performed by the same authors on a patient’s skin biopsy revealed reduced dermal collagen and irregular disrupted collagen fibers, as well as our HF-USG and RCM investigations that disclosed abnormal collagen fibers deposition together with a reduced dermal thickness. Moreover, we recognized an increase of the epidermal thickness likely correlating with the diffuse PoC-like dermatitis, which is probably not related to classical-like EDS type 2. The use of these non-invasive diagnostic techniques may be promising for the investigation of the qualitative and quantitative cutaneous alterations, but further studies including electron microscopy on skin as golden standard of reference on large cohorts of patients are warranted. In our case, we did not perform skin biopsy because of the patient’s will due to psychological reluctance for her important skin fragility with delayed wound healing.

The *AEBP1* variants discovered before our patient’s characterization were all loss-of-function (LOF) mutations (Table 2) and included compound heterozygous variants [(c.1470del; p.Asn490_Met495delins40) and (c.1743C>A; p.Cys581*] in the first individual (P1); a homozygous variant (c.1320_1326del; p.Arg440Serfs*3) in the second individual (P2); a homozygous splice variant leading to skipping of the last 22 bp of exon 13 (c.1630+1G>A) in the two siblings from the third family (P3, P4), and a homozygous nonsense variant (c.917dup; p.Tyr306*) in the two siblings from the fourth family (P5, P6). Hebebrand and coworkers performed the analysis of all *AEBP1* LOF variants reported in multiple databases showing that these are distributed throughout the protein and by using conservative criteria for pathogenic LOF variants (nonsense, frameshift, canonical splice sites, or initiation-codon) these authors estimated a carrier frequency of 1/829 for the gnomAD database. The analysis of CADD scores for all possible missense variants showed a higher predicted deleteriousness for positions close to the discoidin and the MCP-like domains, whereas the unstructured N-/C-terminal parts showed lower scores. The high deleteriousness scores observed for missense variants within these domains cite evidence in support of additional mutational mechanisms leading to aberrant function and the authors thus argued that the relatively low estimated carrier frequencies could be significantly higher if missense variants contribute to a comparable fraction of disease variants [17]. The present c.1925T>C; p.(Leu642Pro) homozygous variant disclosed within the MCP-like domain of the protein corroborates this hypothesis, since it represents the first likely pathogenic *AEBP1* missense substitution (ACMG class 4) associated with classical-like EDS type 2. The variant is predicted *in silico* to affect the tertiary structure of the protein by disrupting an α-helix located in a highly conserved domain, thus likely interfering with its function in terms of impaired partner binding capability. Nevertheless, a definite proof of variant’ s causality is lacking, since the effective functional consequences on the ECM organization, particularly of collagens, and on the other not yet well-defined roles of the ACLP protein were not studied, because the patient refused skin biopsy.

## 5. Conclusions

Our findings expand the knowledge of the clinical phenotype of this recently defined autosomal-recessive EDS subtype, provide the first evidence that missense variants contribute to the allelic repertoire of *AEBP1,* and suggest that in the diagnostic process of a cEDS patient this gene should be investigated when a recessive inheritance is compatible and no causal variant is identified in the other cEDS genes. Further reports are needed to better characterize the *AEBP1*-related phenotype, define specific clinical criteria that might facilitate the differential with the other EDS forms, delineate genotype-phenotype correlations, and collect natural history data for prognostication. Finally, ACLP function needs to be explored more in-depth to provide insights into molecular mechanisms involved in the pathophysiology of *AEBP1*-related EDS that may represent a starting point for identifying potential therapeutic options.

## Figures and Tables

**Table 1:** Epidermal and dermal thickness of patient’ s forearm evaluated by high-frequency ultrasonography (HF-USG) compared to 10 healthy individuals.

	Dorsal Forearm		Volar Forearm	
	Patient	Controls (mean ± SD)	Patient	Controls (mean ± SD)
**Epidermal thickness (µM)**	172	121 ± 22	141	102 ± 12
**Dermal thickness (µM)**	570	1108 ± 320	289	983 ± 205

**Table 2:** Summary of clinical features of individuals with autosomal recessive variants in *AEBP1*

Citation	Present patient	P1*	P2*	P3*	P4*	P5*	P6*
**Sex**	female	male	male	female	male	female	male
**Ethnicity**	white	white	white	Middle Eastern	Middle Eastern	white	white
**Age at evaluation**	53y	35y	33y	12y	24y	39y	38y
***AEBP1* variant(s) (NM_001129.4)**	c.1925T>C homozygous	c.1470del, c.1743C>A compound heterozygous	c.1320_1326del homozygous	c.1630+1G>A (r.1609_1630del) homozygous	c.1630+1G>A (r.1609_1630del) homozygous	c.917dup homozygous	c.917dup homozygous
**Protein change (NP_001120.3)**	p.(Leu642Pro)	p.(Asn490_Met495delins40), p.(Cys581*)	p.(Arg440Serfs*3)	p.(Val537Leufs*31)	p.(Val537Leufs*31)	p.(Tyr306*)	p.(Tyr306*)
**Joint hypermobility (BS)**	+ (5/9)	+ (8/9)	+ (8/9)	+ (8/9)	+ (NA)	+ (6/9)	+ (2/9)
**Dislocations/ Subluxations**	left ankle, knees, shoulders, elbows	hip, right distal radioulnar joint	hip (congenital), shoulders	hip, knees, ankles shoulders, interphalangeal joints	hips, knees and ankles	wrist, mandibular and distal radioulnar joints	ankles, knees, clavicula
**Foot deformities**	pes planus, hallux valgus	pes planus, hallux valgus, hammer toes	pes planus, hallux valgus, hammer toes	pes planus, hallux valgus, hammer toes	pes planus, hallux valgus, toe deformities	pes planus, hallux valgus, sandal gap	hindfoot deformity, sandal gap
**Extensive skin hyperextensibility**	+	+	+	+	+	+	+
**Delayed would healing (abnormal scarring)**	+ (widened atrophic scars)	+ (widened atrophic scars)	+ (widened atrophic scars, keloids)	+ (widened atrophic scars, keloids)	+ (widened atrophic scars)	+ (widened atrophic scars)	+ (widened atrophic scars)
**Redundant skin**	+ old-aging appearance	+ old-aging appearance	+	+	+	+ old-aging appearance	+ old-aging appearance
**Easy bruising**	+	+	+	+	NA	+	+
**Prominent chest superficial veins**	-	NA	+	NA	NA	+	+
**Hernia**	umbilical surgically treated	-	large ventral surgical hernia	umbilical, ventral, inguinal	NA	+	-
**Genitourinary abnormalities**	-	cryptorchidism surgically corrected	-	-	-	-	cryptorchidism surgically corrected
**Gastrointestinal abnormalities**	-	motility issues	bowel rupture	-	-	NA	NA
**Vascular abnormalities**	peripheral artery disease, varicose veins	MVP	MVP, mildly dilated aortic root, bilateral carotids stenosis, aortic dilation requiring surgery	-	-	MVP, circular pericardial effusion	varicose veins
**Dentition**	Pyorrhea, complete dental loss at age 14	retention of a single baby tooth	-	abnormal dental alignment	abnormal dental alignment	-	-
**Citation**	Present patient	P1*	P2*	P3*	P4*	P5*	P6*
**Sex**	female	male	male	female	male	female	male
**Ethnicity**	white	white	white	Middle Eastern	Middle Eastern	white	white
**Age at evaluation**	53y	35y	33y	12y	24y	39y	38y
**Facial dysmorphisms**	high palate, elongated uvula	-	micrognathia	bilateral ptosis webbed neck, sagged cheeks large ears, narrow palate	bilateral ptosis webbed neck, sagged cheeks large ears, narrow palate	-	-
**Skeletal anomalies** **(MRI findings)**	femoral osteopenia, T10 vertebral deformity, scoliosis, lumbar spine rectilinization with marked degenerative arthritis	severe osteopenia of hips (mild disc bulging at the C4-5 and C7-T1 levels)	hip replacement for severe osteopenia, upper thoracic scoliosis with degenerative disease and facet arthrosis of spine (empty sella)	skull with ‘copper beaten’ appearance, severe osteopenia, narrowing of the interpedicular distance of the lumbar spine distally, short and squared iliac bones, remodeled long bones of the lower extremities	severe osteopenia	progressive kyphosis, scoliosis, arachnodactyly, positive wrist and thumb signs, degeneration of the discus ulnaris orthopedically treated	kyphoscoliosis, arachnodactyly, positive wrist and thumb signs, mild pectus excavatum
**Other**	hypotonia, delayed motor development, multiple papules (diffuse PoC-like dermatitis, alopecia, patellar instability surgically treated, rotator cuff disease surgically treated, epitrochleitis, subacromial shoulder impingement, hypotrophy of the scapular girdle, gonarthrosis, chronic fatigue, spheroids, piezogenic papules, myopia	delays in walking and acquisition of fine motor skills, impaired temperature sensation, keratoconjunctivitis sicca, piezogenic papules	elbow bursitis, piezogenic papules, sacral dimple, hypertriglyceridemia	hypotonia, diabetes mellitus, cellulitis	NA	alopecia, skin striae	strabismus surgically treated, myopia, astigmatism

*Patients reported by Alazami et al., 2016 [15], Blackburn et al., 2018 [16], and Hebebrand et al., 2018 [17]. P1: A-II:1;P2: B-II:1; P3:C-IV:6; P4: C-IV:4 according to [16]; P5: D-II:1; P6: D-II-2 according to [17]. Abbreviations: + present, - absent, NA not available, MVP mitral valve prolapse

## Supplementary Materials

The following are available online at http://www.mdpi.com/2073-4425/10/2/135/s1, Table S1. Primers.
